# Pre-existing Alloreactive T and B Cells and Their Possible Relevance for Pre-transplant Risk Estimation in Kidney Transplant Recipients

**DOI:** 10.3389/fmed.2020.00340

**Published:** 2020-07-21

**Authors:** Gonca E. Karahan, Frans H. J. Claas, Sebastiaan Heidt

**Affiliations:** Department of Immunohematology and Blood Transfusion, Leiden University Medical Center, Leiden, Netherlands

**Keywords:** HLA-specific memory, alloantibodies, alloimmune response, T cell-mediated rejection (TCMR), antibody-mediated rejection (ABMR)

## Abstract

In allogeneic transplantation, genetic disparities between patient and donor may lead to cellular and humoral immune responses mediated by both naïve and memory alloreactive cells of the adaptive immune system. This review will focus on alloreactive T and B cells with emphasis on the memory compartment, their role in relation to kidney rejection, and *in vitro* assays to detect these alloreactive cells. Finally, the potential additional value of utilizing donor-specific memory T and B cell assays supplementary to current routine pre-transplant risk assessment of kidney transplant recipients will be discussed.

## Introduction

Immunological memory, the capacity to mount a rapid and robust immune response when a given antigen is re-encountered, protects individuals against a wide range of pathogens following infections or vaccinations (1). Both cellular and humoral adaptive immune responses contribute to this long-lasting protection, making long-lived memory T and B cells the central players in immunological memory. These cells are generated by clonal expansion of a subset of antigen-specific naïve cells during primary immune responses, and can persist over decades after the pathogen has been eliminated (2–4). Although recent evidence suggests that some features of immunological memory exist in innate immune cells, memory T and B cells of the adaptive immune system will be the focus of this review (5).

While being extremely effective in protecting individuals against pathogens, memory T and B cells can sabotage successful allogeneic transplantations by eliciting vigorous alloimmune responses to donor antigens. Alloreactive memory T and B cells may arise as a result of exposure to allogeneic human leukocyte antigens (HLA) through blood transfusions, pregnancies or previous transplantations. Interestingly, individuals who have never been exposed to alloantigens may as well harbor alloreactive memory as a result of heterologous immunity (6, 7).

Advances in immunosuppressive drugs have led to a dramatic reduction in acute rejection rates resulting in significant improvements in short term allograft survival. However, lack of improvement of long-term graft survival remains a major problem in kidney transplantation. Furthermore, repeat transplantation candidates, as well as those with a history of pregnancy and multiple blood transfusions who have broad HLA sensitization tend to accumulate on transplant waitlists. These patients are not only difficult to pair with a crossmatch negative donor in order to be transplanted but are also more susceptible to develop T cell and/or antibody-mediated acute and chronic rejection unless special allocation algorithms or desensitization treatments are used (8–12).

In the setting of kidney transplantation, the current practice of immunological pre-transplant risk assessment mainly focuses on the presence or absence of plasma cell-derived donor HLA directed antibodies (DSA) in the serum of patients, in addition to HLA matching between the patient and donor (13, 14). This strategy, however, ignores the potential contribution of alloreactive memory cells to graft rejection (15–19). In this review, we will focus on alloreactive (memory) cells and their possible contribution to rejection episodes primarily in the setting of kidney transplantation, and provide an overview of *in vitro* assays to detect alloreactive memory T and B cells. Furthermore, we will elaborate on the potential use of these assays in pre-transplant immunological risk assessment of kidney transplant recipients.

## Contribution of Alloreactive T and B cells to Graft Rejection

### Alloreactive T Cells

**Figure 1:**
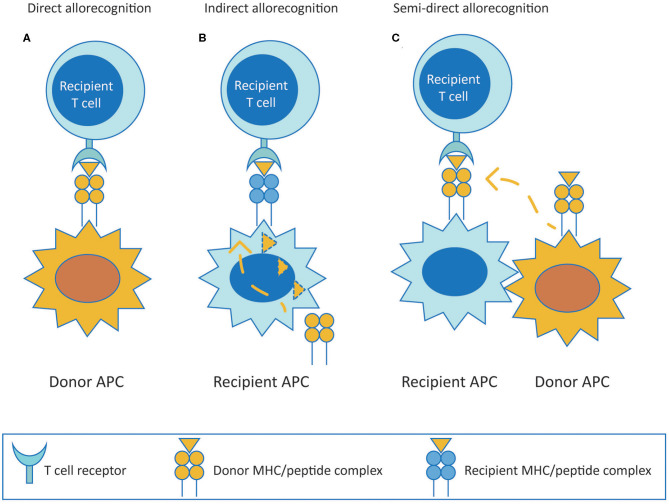
T cell allorecognition pathways. **(A)** (Direct pathway) Recipient T cells recognize intact donor alloantigens on the surface of donor APC. **(B)** (Indirect pathway) Recipient T cells recognize processed donor allogeneic peptides presented on the context of self MHC antigen by recipient APC. **(C)** (Semi-direct pathway) Recipient T cells recognize intact donor MHC acquired by recipient APC. MHC, major histocompatibility complex; APC, antigen presenting cell.

Alloreactive T cells are considered to be the central players in mediating allograft rejection. They contribute to both acute and chronic rejection depending on the pathway utilized to recognize donor antigens (both major and minor histocompatibility antigens). T cells recognize alloantigens through the direct, indirect or semi-direct pathway (Figure 1). The direct pathway of T cell recognition is unique to allogeneic transplantation, and involves both CD4 and CD8 T cells of the recipient recognizing intact allogeneic major histocompatibility complex (MHC) antigens class II and I, respectively, expressed on the surface of donor cells (Figure 1A). This pathway of allorecognition is considered to be short-lived, especially for HLA class II, due to the limited life-span of donor dendritic cells migrating to lymphoid tissues of the recipient to initiate the immune response. Therefore, the direct pathway T cells are considered to be the predominant mediators of acute cellular rejections in the early post-transplantation period, although MHC expressed on graft parenchyma may as well directly activate T cells at any time after transplantation, contributing to long term injury (20–23).

In comparison to conventional T cell responses to protein antigens, the direct pathway alloimmune response is stronger, likely due to the high frequency of direct pathway alloreactive T cells (24). This allows for measurement of direct pathway alloimmune responses *in vitro* without the need for priming in mixed lymphocyte reactions (MLR). T cell alloimmune responses measured *in vitro* involves CD4 and CD8 T cells with contributions both from naïve and memory T cell fractions (25, 26). Between 1-10% of circulating T cells in humans are known to be alloreactive as tested by traditional assays (27, 28). Recently, using high throughput sequencing in combination with MLR in healthy individuals, Emerson et al. observed an average of 14,000 alloreactive T cell clones in each experiment they performed. Strikingly, antigen-experienced memory T cell clones made up to 60% of the alloreactive T cell repertoire (29). In addition, the alloreactive memory T cell repertoire could be detected at similar clonal frequencies in a later time point sample when the same allogeneic donor was used for stimulation in MLR, indicating their persistence in circulation. Presence of alloreactive memory T cells in individuals who have never been exposed to alloantigens is supportive for a role of heterologous immunity by which T cells generated in response to infectious or environmental antigens can cross-react with allogeneic MHC antigens (30). Indeed, cross reactivity of virus-induced memory T cells with allogeneic HLA has been shown to be common (7). A classic example of cross reactivity of virus-induced memory T cells with alloantigens is that of HLA-B^*^08:01 bearing patients who have been exposed to Epstein-Barr virus (EBV) infection showing cross-reactivity to allogeneic HLA-B^*^44:02 (6, 31). Cross-reactivity of virus-induced T cell receptors (TCR) with alloantigens could be of clinical relevance because they have been shown to directly recognize donor MHC and cause allograft rejection in murine studies. However, a significant impact on transplantation outcome in humans has not been shown so far (32, 33).

The indirect pathway is analogous to adaptive T cell responses mounted to common protein antigens, and involves alloreactive T cells of the recipient recognizing allogeneic MHC class I or class II as processed peptides presented in the context of self MHC class II (Figure 1B). Indirect pathway alloreactive CD4 T cells can provide help to induce cytotoxic CD8 T cells and are known to be the only cells that can provide help to alloreactive B cells (34–36). The indirect pathway of T cell allorecognition is considered to be long-lasting and particularly important in the development of chronic allograft rejection because of exclusive cognate help provided by indirect CD4 T cells to alloreactive B cells leading to alloantibody production. Indirect allorecognition can also apply to alloreactive CD8 T cells through cross-priming whereby antigen presenting cells (APC) present alloantigenic peptides in the context of class I molecules (37). However, indirect pathway CD8 T cells have been shown to have no role on alloimmune response to vascularized cardiac allografts in murine models, possibly because CD8 T cells require direct contact with target cells to exert their cytolytic effects (38). Additionally, indirect alloresponses against minor histocompatibility antigens may also occur. However, in comparison to the enormous polymorphism of MHC, indirect allorecognition of minor antigens appears to be less relevant (39).

Finally, in the semi-direct pathway, recipient T cells recognize intact allo-MHC similar to direct way of allorecognition but on the surface of self APC that have acquired allo-MHC by various means including cell to cell contact or exosomes, suggesting a role for both direct and indirect allorecognition pathways in chronic alloimmune responses (Figure 1C) (40, 41).

### Alloreactive B Cells

Humoral alloimmunity can lead to antibody mediated rejection (ABMR), of which the chronic form is the leading cause of graft loss in kidney transplantation (42). Interaction between alloreactive T and B cells plays a key role in the generation of full-blown humoral alloimmune responses. Help from indirect pathway CD4 T cells is essential for generating antibodies against HLA, as with conventional protein antigens. Typically, when a naïve B cell ligates a protein antigen via its B cell receptor (BCR) in secondary lymphoid organs, it internalizes the antigen by receptor mediated endocytosis and then migrates to the interface between the B cell follicles and the T cell zone of the lymphoid tissue. At the T-B cell border of the lymphoid tissue, activated B cells present the processed peptide in the context of self MHC class II to cognate CD4 T cells in the presence of CD40-CD154 ligation (43). Activated B cells receiving help from CD4 T cells at the T-B cell border can either form an extrafollicular response and become short-lived plasma cells or go back to B cell follicles and initiate germinal center reactions (44). In the pre-germinal center period, class switching to IgG isotype may occur whereas somatic hypermutations are not observed and antibodies of the extrafollicular foci are known to be low/moderate affinity antibodies (45). Some of the activated B cells in the extrafollicular foci may give rise to germinal center independent memory B cells. Within germinal centers, B cells undergo somatic hypermutation to increase the affinity of their BCRs for the antigen. B cells that recognize the antigen presented by follicular dendritic cells in germinal centers internalize, process and present the peptides to follicular T helper cells (Tfh) (46). In germinal centers, B cells compete for limited availability of Tfh interactions of which are critical for high affinity B cells to be selected further (47). Those B cells that have an improved affinity for the antigen are selected to leave the germinal centers either as isotype switched memory B cells or plasma cells producing high affinity isotype switched antibodies (IgG, IgA, and IgE). While B cells with highest affinity antigen receptors are preferentially recruited to the plasma cell pool, B cells with less high BCR affinity may be selected for the memory B cell pools. Less stringent selection criteria for memory B cells generates a more diverse memory B cell repertoire compared to long-lived plasma cell pools (48, 49). Some germinal center B cells leave germinal centers before the class switch recombination occurs and may give rise to IgM memory B cells which have the capacity to re-enter germinal centers and give rise to new IgG memory B cells (45).

Following germinal center reactions, plasma cells home to the bone marrow and mucosal tissues to become long-lived plasma cells, whereas memory B cells circulate in their quiescent form until a reencounter with the antigen takes place. Despite the fact that the fate of B cells following germinal centers is extensively investigated in studies focusing on responses against pathogenic or vaccine antigens, it is still unknown what proportion of B cells going through germinal center reactions is committed to become plasma cells or memory B cells. For some viral and vaccine antigens, correlations of memory B cells with serum antibodies are known (3) but it is not clear whether immunization against a certain HLA antigen will always result in generation of both memory and plasma cells and whether the ratio of these cells is different in individuals, per route of immunization, and even per HLA antigen encountered. Likewise, while more diversity has been shown for the memory B cell repertoire in comparison to serum antibodies using high throughput heavy chain variable region sequencing of BCR and serum IgG in the setting of tetanus toxoid vaccination in humans (49), knowledge on memory B cell derived vs. serum HLA antibody profiles is newly emerging with indications suggesting that they are not identical (50–52). The latter is particularly important considering the property of memory B cells to rapidly differentiate into antibody producing cells and to give rise to donor-specific antibody (DSA) responses in patients previously known to have no serum DSA (53). In addition to antigen-specific re-encounter, anamnestic responses can also derive from bystander activation of HLA-specific memory B cells upon infection or vaccination (54). In this context, given that exposure to viral infections shapes the alloreactive T cell repertoire of an individual, we wondered whether this would hold true for B cells as well. However, by testing several virus-specific monoclonal antibodies against HLA and many HLA-specific monoclonal antibodies against viral antigens, we did not detect such a cross-reactivity between viral antigens and HLA for B cells at least at the level of monoclonal antibodies (55).

### T-B Cell Interactions

Interactions between alloreactive T and B cells in secondary lymphoid organs may have implications on the strength and specificity of alloantibody produced. A recent study in a mice heart transplant model, designed to investigate solely the contribution of indirect allorecognition to ABMR in the absence of direct pathway CD8 cytotoxic T cells, showed that when a high number of alloreactive CD4 T cells are present to provide help to B cells, this may lead to extrafollicular alloantibody responses with moderate affinity yet still capable of binding to endothelial cells and activating complement and causing acute ABMR, regardless of the number of allospecific B cells (56). On the contrary, germinal center activity leading to high affinity antibody production and progression to chronic allograft injury was more profoundly influenced by the number of antigen-specific B cells provided that they receive help from Tfh cells (57). Differences in the nature of interactions between alloreactive CD4 T cells and B cells shown in these studies suggest that some *de novo* DSA that disappear early after transplantation may be a product of extrafollicular foci, whereas those that persist may be high affinity DSA produced by long-lived plasma cells and both with the capacity to mediate ABMR.

Conventionally, CD4 T cells and B cells that are specific for different epitopes of the same antigen cooperate through linked recognition in order to generate long-lasting humoral immunity specific for a protein antigen. Interestingly, studies in mice have shown evidence that B cells recognizing one donor MHC could receive help from T cells specific for another allogeneic MHC on the donor organ although the alloantibody levels generated were relatively lower compared to the conventional way of receiving help in this “unlinked help condition” (58). Interestingly, when naïve CD4 T cell help was abolished by co-stimulation blockade targeting CD40/CD154 interactions, interferon-gamma (IFN-γ) producing memory CD4 T cells could still provide help to B cells (59, 60). Hypothetically, in a setting where there are multiple HLA mismatches between the patient and donor and alloreactive T cell activation is independent of alloreactive B cell activation, memory CD4 T cells generated in response to a previous alloantigen may provide help to a naïve B cell leading to the production of a different alloantibody specificity and isotype at least through extrafollicular immune responses (58). This type of immune response in humans could also occur when high frequency alloreactive memory CD4 T cells are present before transplantation as a result of previous alloantigen exposure or heterologous immunity.

## *In vitro* Methods to Detect Alloreactive T and B cells

**Figure 2:**
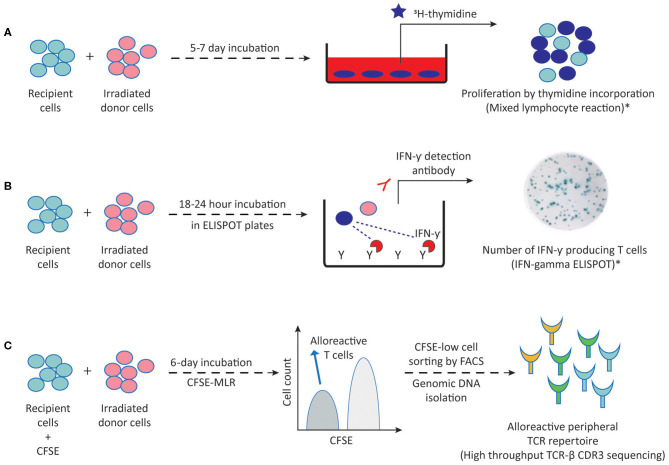
Methods to detect alloreactive T cells. **(A)** Proliferation of direct pathway alloreactive T cells following 5–7 days incubation of recipient cells (responder) with irradiated donor cells (stimulator) can be measured by ^3^H-thymidine incorporation into proliferating cells in MLR. **(B)** Following a 18–24 h MLR in ELISPOT plates, IFN-γ producing direct pathway primed/memory T cells can be visualized by addition of the IFN-γ detection antibody. **(C)** Following a 6-day MLR, CFSE-low dividing direct pathway alloreactive T cells are sorted by FACS and genomic DNA is isolated for high throughput TCR-β CDR3 sequencing. This generates an alloreactive TCR repertoire which can then be compared to the TCR repertoire of the unstimulated patient sample. ^3^H-thymidine, tritiated thymidine; CFSE, carboxyfluorescein succinimidyl ester; MLR, mixed lymphocyte reaction; TCR-β, T cell receptor beta chain; FACS, fluorescence activated cell sorting; CDR3, complementarity determining region-3. Dark blue circles represent alloreactive T cells. *These methods are also being used for detecting indirect pathway alloreactive T cells by replacing irradiated donor cells with donor cell fragments, synthetic peptides or synthetic HLA molecules.

**Figure 3:**
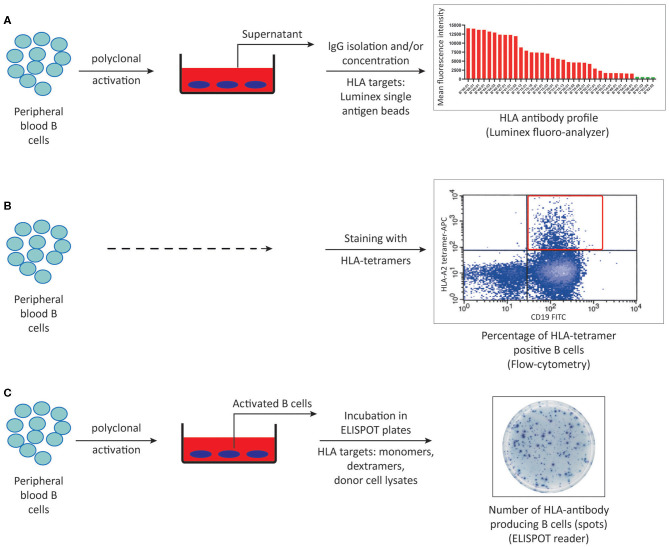
Methods to detect HLA-specific memory B cells. **(A)** Peripheral blood B cells can be polyclonally activated and IgG isolated culture supernatants can be screened for memory B cell derived-HLA antibodies using luminex single antigen bead assays. **(B)** Peripheral blood B cells can be stained with HLA-tetramers using flow cytometry. **(C)** HLA-specific memory B cells can be quantified by ELISPOT assay using either synthetic or donor-derived HLA molecules following *in vitro* polyclonal activation. ELISPOT, Enzyme-linked immunosorbent spot.

The development of *in vitro* methods allowing for accurate, sensitive and reproducible detection and quantification of donor-specific alloimmune responses has long been a challenge in the field of transplantation immunology. Such assays are required not only to detect the sensitization against a potential donor before transplantation, and thus select the patient-donor pair with the lowest risk of alloimmune responses, but also to be able to monitor the ongoing alloimmune response post-transplant with non-invasive methods in order to adjust the immunosuppressive treatments. Currently available methods to detect alloreactive T and B cells are outlined in Figures 2, 3, respectively, and in Table 1.

**Table 1:** Overview of methods to detect alloreactive T and B cells.

	**Method**	**Principle**	**Duration**	**Properties**	**Limitations**
T cells	Mixed lymphocyte reaction (MLR)	Incubation of recipient cells with intact donor cells (direct allorecognition) or fragmented donor cells (indirect allorecognition)	6–7 days	-Proliferation by thymidine incorporation -Frequency calculation by limiting dilution or CFSE-dilution analyses-Cytokine and sCD30 measurement in culture supernatants possible -Measurement of direct and indirect alloreactive T cell responses	-Requirement for donor cells -Too labor intensive for accurate frequency calculation -Bystander proliferation of non-alloreactive T cell clones -Indirect pathway measurement lacks specificity and sensitivity
	IFN-γ ELISPOT	MLR in ELISPOT plates	18–24 h	-Number of IFN-γ producing alloreactive primed T cells -Measurement of direct and indirect alloreactive memory T cell responses	-Low predictive capacity for clinical outcome at individual patient level -Indirect pathway measurement lacks specificity and sensitivity
	TCRβ-CDR3 sequencing	CFSE-MLR followed by high throughput sequencing	6–7 days	-Longitudinal monitoring of changes in the alloreactive clone size and diversity-Currently only used to measure direct alloreactive T cell responses	-Requirement for pre-transplant MLR -Current assay costs
B cells	B cell culture supernatant analysis	*In vitro* polyclonal activation of peripheral blood B cells	10 days	-Qualitative assay detecting memory-B cell derived HLA antibodies in supernatants -Enables direct comparison of serum and memory HLA antibody profiles -Easy-to-apply and standardize in all HLA labs -High sensitivity and specificity	-Does not allow for quantification of HLA-specific memory B cells -Long culture time
	HLA-specific tetramer staining	Binding of HLA tetramers to B cell receptors	1 days	-Quantitative assay providing percentage of HLA-tetramer positive B cells -Straightforward method using flow-cytometry	-Non-specific tetramer binding to B cells -Not all HLA-tetramer positive B cells secrete antibodies -Requires a second step of cell sorting and culturing -Limited resources for synthetic HLA class II molecules
	HLA-ELISPOT	*In vitro* polyclonal activation of peripheral blood B cells followed by binding of IgG to synthetic or lysate HLA	6–7 days	-Fully quantitative assay -Each HLA-antibody producing memory B cell can be visualized as spots	-Labor intensive -Difficult to test against multiple donor HLA mismatches when using synthetic HLA molecules -Limited resources for synthetic HLA class II molecules -Requirement for donor cells when lysate is used -Difficult to standardize for clinical use

*TCRβ-CDR3, T cell receptor-beta chain, complementarity determining region 3; CFSE, carboxyfluorescein succinimidyl ester; sCD30, soluble CD30*.

### Direct Pathway T Cells

T cells with direct alloreactive capacity have been shown to play a predominant role in acute rejection occurring early after transplantation. Mixed lymphocyte reaction (MLR) stands as the traditional and most commonly used method to measure donor HLA class II specific T cell alloreactivity and forms the basis for complementary approaches to further assess the frequency and diversity of the alloreactive T cell repertoire. The MLR is based on culturing patient peripheral blood mononuclear cells (PBMC) or T cells as responder cells with irradiated allogeneic donor cells (stimulator) for 5–7 days (Figure 2A). For an accurate donor-specific response calculation, autologous cells of the patient against irradiated autologous targets, fully HLA-mismatched third-party cells as stimulators, and a non-specific stimulation of patient T cells (e.g., PHA or Concavalin-A) as a positive control should preferentially be included. Approximately 18–24 h before the end of the MLR, cells are pulsed with radioactive thymidine to determine the level of proliferating patient T cells. Early studies have defined the relative donor-specific response rate either as hypo-responsiveness or as hyper-responsiveness in kidney transplant recipients and related these responses to good graft function or acute rejection within the first year after transplantation, respectively (61, 62). However, limited predictive value of the MLR for acute rejection has been reported since (63, 64).

Measuring proliferation alone in MLRs does not give accurate information on the effector functions or the frequency of donor-specific alloreactive cells. While tedious limiting dilution assays can be used to quantify both cytotoxic T lymphocyte precursors and IL-2 producing helper T cells (65, 66), a carboxyfluorescein succinimidyl ester (CFSE)-based MLR can easily help to dissect precursor frequencies of dividing alloreactive CD4 and CD8 T cells. Measuring CFSE dilutions in MLR using flow cytometry provides a better estimation of precursor frequencies, since CFSE dilution indicates the number of times the cells have divided throughout the entire MLR rather than giving a snapshot of proliferation over a period of time in culture as thymidine incorporation does. Macedo et al. showed that CD4 and CD8 T cells proliferated equally in response to allo-stimulation with average frequencies of 4% of total T cells by using a CFSE-MLR in combination with additional cell markers (25). However, precursor frequency calculations should be interpreted carefully because of the potential bystander proliferation of non-alloreactive clones in MLRs.

Both naïve and memory T cells contribute to alloimmune responses (25, 26, 30). Given that memory T cells are primed to respond to alloantigens more rapidly in comparison to their naïve counterparts, Heeger and colleagues developed an enzyme-linked immunosorbent spot (ELISPOT) assay in which IFN-γ producing alloreactive primed/memory T cells could be detected and quantified upon 18–24-h MLRs (Figure 2B) (67, 68). Using the IFN-γ ELISPOT assay, several researchers found a correlation between pre-transplant alloreactive memory T cell frequencies and subsequent acute rejection within the first year of kidney transplantations, independent of HLA antibodies or HLA matching (16, 67–69). These results, in combination with efforts to bypass the need for donor cells inspired its utilization as a screening tool against a panel of allogeneic stimulator cells (panel of reactive T cells-PRT) in pre-transplant risk assessment of kidney transplant recipients, analogous to panel reactive antibody testing (70–72). Using this PRT approach, Poggio et al. showed that 63% of pre-transplant PRT-positive patients experienced acute cellular rejection in a small cohort of 30 transplant recipients (72). However, a recent study performed on 168 consecutive kidney transplant recipients concluded that pre-transplant IFN-γ ELISPOT positivity correlated with biopsy proven acute rejection only when performed in a donor-specific manner and not against a panel of surrogate donors (17). While a clear correlation between donor-specific pre-transplant IFN-γ ELISPOT and acute rejection risk could not be established in a multicenter clinical trial of 176 kidney transplant recipients (73), Crespo et al. found a strong correlation between IFN-γ ELISPOT positive patients and early acute cellular rejections only in a subset of patients not receiving T lymphocyte depleting induction therapy (74). The latter finding is particularly interesting considering that after T lymphocyte depleting therapies, homeostatic proliferation favors memory T cell population. Yet, lower memory T cell frequencies may be due to the effect of other immunosuppressive agents used in combination with T cell depleting agents such as calcineurin inhibitors (75, 76). Moreover, although a harmony on standardization and validation of IFN-γ ELISPOT assays between different laboratories has been shown (77, 78), the IFN-γ ELISPOT approach suffers from predicting outcomes for individual patients despite correlations with outcome at the population level (79).

CD30 is considered to be a marker to identify a subset of alloreactive T cells producing IFN-γ *in vitro* (80). Several studies showed the pre- and post-transplant prognostic value of serum soluble CD30 (sCD30) levels in predicting graft survival and efficacy of immunosuppressive treatments in kidney transplant recipients (81, 82). More recently, Velasquez et al. have shown the predominant dependence of sCD30 release on memory CD4 T cells in MLR culture supernatants, suggesting that high serum sCD30 levels in kidney waitlist patients could be attributed to active memory T cell responses (83). While this may be an explanation for the significantly lower 3-year graft survival rate in patients with simultaneous pre-transplant DSA and sCD30 positivity in comparison to those with DSA only (84), lack of direct biological link between deleterious DSA and sCD30 as well as the absence of evidence on whether sCD30 release is correlated with donor-specific T cell proliferation render sCD30 to be a non-specific marker of immune activation.

Recently, with the advent of T cell receptor deep sequencing in combination with CFSE-MLR, the size and diversity of the donor-specific T cell repertoire of a particular patient can be monitored before and after transplantation (29, 85). This can be achieved by fluorescence activated cell sorting (FACS) of alloreactive CD4 and CD8 T cells of the patient based on CFSE-MLR, isolating genomic DNA from the divided alloreactive populations as well as from unstimulated patient cells and comparing TCR-beta chain complementarity determining region 3 (CDR3) using high throughput sequencing (Figure 2C). Since each individual T cell clone has a distinct CDR3 sequence, such an approach allows for generation of a “catalog” demonstrating alloreactive clone size and diversity for a patient. Using this approach in a cohort of combined kidney-bone marrow transplant patients, Morris et al. found a decrease in circulating pre-transplant donor-reactive CD4 and CD8 T cell clones in tolerant patients, whereas such a reduction was not observed in non-tolerant patients on immunosuppression (85). The same approach can be used for monitoring changes in frequencies of alloreactive naïve and memory T cells by mapping MLR-expanded alloreactive T cell fractions to the sorted naïve or memory fractions of the unstimulated population (26). However, cell numbers, as well as low frequency clones may be a limiting factor in practice. Even so, TCR-beta CDR3 sequencing stands out to be a highly promising method by which post-transplantation fate of donor-specific direct pathway alloreactive T cell clones can be monitored in comparison to pre-transplant “catalog” of a patient without the need for further functional assays.

### Indirect Pathway T Cells

Several studies suggested a role for indirect pathway alloreactive T cells in both acute and chronic allograft rejection of kidney, heart and lung transplant patients using the MLR-based methods originally applied for direct allorecognition (20, 21, 23, 86–90). Since indirect allorecognition is caused by the activation of T cells by processed alloantigens presented as peptides in the context of self MHC class II, properly fractionated cells can theoretically be used as the source of antigens, given the assumption that no intact HLA molecules are remaining (21, 23). Increased indirect pathway responses in kidney transplant recipients experiencing chronic rejection were found when cytoplasmic membrane proteins from donor cells were used to stimulate patient T cells in MLR (21). Using a similar approach in IFN-γ ELISPOT assays, a significant correlation between proteinuria and indirectly activated IFN-γ secreting primed/memory T cells in patients >2 years after kidney transplantation was shown (23). While cellular fragments likely provide a proper representation of the donor alloantigen repertoire, controls should be taken along in order to exclude involvement of direct/semidirect pathway recognition which may activate patient T cells due to residual intact antigens in these donor cell fragment preparations. On the other hand, lack of standardization in preparation of cellular fragments may lead to poor reproducibility. To overcome this issue, synthetic peptides produced in a standardized way, corresponding to polymorphic domains of mismatched donor MHC class I and II as well as peptides deriving from oligomorphic domains can be used. However, one should be aware of the possibility of the recognition of neo-epitopes which do not exist *in vivo* on peptides by recipient T cells and include peptides from self HLA as controls (91–94). Recently, HLA monomers were suggested as tools to study indirect alloreactivity, but the requirement for very high concentrations of these monomers in order to induce a reproducible indirect T cell response limited their use as the antigen source in this setting (95, 96).

Methods to detect indirect pathway are particularly important since only indirect pathway CD4 T cells can provide help for alloreactive B cells to produce alloantibodies, as shown in animal models (35, 36). On the other hand, currently there is no reliable *in vitro* assay to detect indirect pathway CD4 T cells mainly due to the problems related to the antigen source used to activate these T cells (specificity) and the low frequency of indirectly recognizing T cells (sensitivity) (97).

### HLA-Specific Memory B Cells

Humoral alloimmune responses to mismatched donor HLA can be detected as circulating HLA antibodies or as HLA-specific memory B cells. Serum antibodies are produced by long-lived plasma cells residing in the bone marrow, whereas memory B cells are quiescent cells that continuously circulate between the secondary lymphoid organs and peripheral blood. In kidney transplantation, pre-existence or *de novo* development of IgG isotype DSA has been clearly shown to be associated with both acute and chronic ABMR and poor graft survival (11, 12, 98). Given that memory B cells may replenish the pool of long-lived plasma cells and may harbor a more diverse repertoire than serum (99), current immunological risk assessment based on detection of circulating HLA antibodies may be incomplete due to the lack of information on the possible presence of donor-reactive memory B cells (53).

Unlike plasma cells that spontaneously produce antibodies, memory B cells need to be stimulated either by re-encountering the same antigen they have seen in primary responses or via bystander activation in a non-antigen specific manner to become antibody producers (54, 100). In order to be detected as antibody secreting cells *in vitro*, memory B cells need to be polyclonally activated (101). While several protocols exist to activate B cells *in vitro*, it is critical that the polyclonal activation cocktail does not induce isotype switching in the naïve B cell population allowing for accurate detection of antibodies deriving from only pre-existing memory B cells. These cocktails stimulate B cells either by ligation to the BCR or Toll-like receptors and/or costimulatory molecules in combination with B cell cytokines and growth factors (102, 103). The ability of *in vitro* activated B cells from alloantigen exposed individuals to secrete HLA-specific antibodies was first demonstrated by our group, paving the way to the development of HLA-specific memory B cell assays (104). In a few studies screening for HLA antibodies in culture supernatants, neat culture supernatants were 10-fold concentrated in order to increase the detectability of the HLA antibodies. A recent method developed by our group in which IgG isotype of antibodies are isolated from culture supernatants resulted in ~20% increase in detectability of HLA-specific memory in alloantigen exposed individuals in comparison to 10-fold concentration (Figure 3A) (52). In a small cohort of 13 alloantigen exposed individuals, we showed that HLA antibody profiles derived from serum and memory B cells were not identical. Unlike the sequencing of BCR and serum IgG in the setting of tetanus toxoid vaccination (49), a broader serum repertoire was the most commonly observed profile, followed by a complete overlap of HLA antibody specificities in serum and culture supernatants. Interestingly, 10% of the HLA antibody specificities were only found in the memory B cell compartment, which may be of clinical relevance. In a cohort of 20 kidney patients transplanted across a DSA barrier we found that concurrent presence of donor-specific memory and serum DSA pre-transplantation was associated with higher 1-year incidence of ABMR and more severe microvascular inflammation in allograft biopsies (19). While supernatant analysis of IgG isolated cultures is an easy and sensitive way of profiling memory B cell derived HLA antibodies in addition to its potential in clinical use, it does not allow for quantification of HLA-specific memory B cells.

One way to quantify HLA-specific memory B cells is based on the ability of BCR to bind to synthetic HLA molecules and can be achieved by flow cytometric analysis upon staining B cells with HLA-tetramers (Figure 3B) (105, 106). Tetramer staining by flow cytometry requires a second step of sorting and culturing to confirm the antibody production capacity of these tetramer positive cells. Considering that not all tetramer positive cells will turn out to be antibody producers, in combination with non-specific binding of BCRs to HLA tetramers, quantification of HLA-specific memory B cells by tetramer positivity alone should be interpreted cautiously.

HLA-ELISPOT assays combine the ability of B cells to produce antibodies upon *in vitro* polyclonal activation with BCR's ability to bind to synthetic HLA molecules, thereby enables detection and quantification HLA-specific memory B cells (Figure 3C). Importantly, since ELISPOT assays purely rely on the capacity of activated B cells to produce antibodies *in vitro*, viability of the stimulated cells at the end of pre-culture phase plays an extremely important role in the quality and accuracy of the ELISPOT results. Hence, in contrast to supernatant analysis favoring longer culture periods up to 10–12 days to achieve the highest IgG concentrations in the culture supernatants, pre-culture phase of ELISPOT assays are preferred to be 6–7 days in order to have highest number of viable cells producing antibodies. Following 6–7 days of culturing, these polyclonally activated B cells are transferred to special ELISPOT plates coated with anti-IgG antibodies to capture the IgG isotype of antibodies produced by polyclonally activated memory B cells. In the second phase of the assay (visualization), antibody fingerprints captured on the filter of ELISPOT plates can be visualized as a single spot representative of one HLA antibody producing cell, using either synthetic (monomeric or multimeric) or donor-lysate derived HLA molecules serving as detection matrix (18, 107–110). Since HLA-specific memory B cells assays are not standardized assays such as the commercially available HLA-antibody detection kits, it is critical to include positive and negative controls to assure a certain standard in each phase of ELISPOT assay. In this regard, performing total IgG ELISPOT assays with every HLA-specific memory B cell ELISPOT is necessary in order to confirm that polyclonal activation indeed gave rise to antibody producing cells at the end of the pre-culture phase. Regarding the visualization phase, one such control would be including HLA-specific hybridoma cells to assure that the HLA target used as the detection matrix, regardless of its source, can give rise to a positive signal (111, 112). Similarly, since no HLA-specific antibody production is expected to occur against self, autologous controls using self HLA as the detection matrix can serve as a negative control in ELISPOT assays. Furthermore, when analyzing memory B cell assays, one should take into account the frequency of spots in autologous controls, if any, as well as the total IgG production capacity of the polyclonally activated cells in order to accurately define the frequency HLA-specific memory B cells per IgG producing cells (110). Using ELISPOT methods, increased frequencies of HLA-specific memory B cells in the absence of serum DSA pre-transplantation and at the time of ABMR in kidney transplant patients have been reported (18, 113). While these results demonstrate the clinical relevance of HLA-specific memory B cell testing in addition to serum DSA analysis, complexity of the ELISPOT method in addition to the labor intensity necessary for accurate performance makes clinical utility of HLA-ELISPOT assays difficult. It is important to note that inherent to all antigen specific B cell detection methods performed on peripheral blood samples, HLA-specific memory B cells may not be circulating at the time of sampling and instead can be residing in secondary lymphoid organs, precluding their detectability.

## A place for HLA-Specific Memory T and B cell Assays in Pre-transplant Risk Assesment?

Alloreactive immune memory contributes to early allograft rejection that is difficult to block or inhibit, rendering a subset of kidney transplant recipients at risk for developing ABMR or T cell mediated rejections (TCMR) (14). To further improve pre-transplant risk estimation in these patients, it would be appropriate to implement T and B cell memory assays as additional tools.

Donor-specific alloreactive T cells are the central players in allograft rejection and a relatively large fraction of this repertoire consists of memory T cells (29). In this regard, IFN-γ producing alloreactive T cell frequencies of the direct pathway as measured by ELISPOT could be a good assay candidate to be used in practice. However, the actual predictive value per individual patient needs to be determined by well-characterized large scale studies, such as the BIO-DrIM consortium (114). Moreover, the need for donor cells in addition to the fact that IFN-γ ELISPOT can only be used to screen patients for living-donor transplantations due to the time consumed to perform the assay are the obstacles in the way of its use.

Currently, high throughput TCRβ CDR3 sequencing of alloreactive direct pathway T cells selected upon MLR offers the possibility to monitor the changes in the frequency of donor-specific T cell clones after transplantation. Application of this approach in larger cohorts may identify the dominant T cell clones leading to rejection or tolerance, yet still requirement for pre-transplant MLR is still a limitation for clinical use. Nonetheless, modification of the TCRβ CDR3 sequencing for indirect pathway T cells is desired to improve our understanding of the mechanisms involved in chronic allograft rejection. Since indirect allorecognition plays a central role in graft rejection and tolerance, the ability to measure indirect allorecognition accurately is an absolute requirement to understand the evolution of alloimmune responses and aid-in application of tailor-made immunosuppressive approaches.

Recently, high throughput sequencing technology has been applied to monitor changes in the B cell repertoire before and after desensitization treatments in highly sensitized individuals with the aim to be able to distinguish responders to treatment from non-responders however no dominant B cell clone that may influence the response to treatment was found (115). In a recent study involving a small cohort of pediatric kidney transplant recipients, B cell repertoire was longitudinally assessed using high throughput BCR sequencing. Patients who experienced rejection had higher B cell diversity before transplantation which decreased post transplantation in comparison to those who did not progress to rejection or chronic injury (116). While BCR heavy chain variable region CDR3 analyses in these studies provide information on the bulk B cell repertoire, HLA-specific IgG and memory B cell repertoires can also be delineated using a similar approach in combination with HLA-specific B cell and antibody sorting. An ideal HLA-specific memory B cell assay, however, should be easily applicable in all HLA laboratories in a standardized way and should preferentially be compatible to current practice of serum HLA antibody analysis.

## Conclusion

Currently, no single assay is capable of capturing all aspects of alloreactive cellular and humoral immune responses. Therefore, combinations of different assays in addition to current practice should be used to have a complete picture of the alloimmune response. Inherent to all the above-mentioned assays sampling peripheral blood, responses from circulating lymphocytes detected in these assays may not be reflective of those infiltrating the donor organs or some responses may be undetected because donor-specific cells might be residing within the graft or in secondary lymphoid organs. Nevertheless, in order to better understand the predictive values of these assays for individual patients, studies with larger cohorts are warranted.

## Author Contributions

GK, FC, and SH wrote the manuscript. All authors contributed to the article and approved the submitted version.

## Conflict of Interest

The authors declare that the research was conducted in the absence of any commercial or financial relationships that could be construed as a potential conflict of interest.
